# Computational Protocol for the Identification of Candidates for Radioastronomical Detection and Its Application to the C_3_H_3_NO Family of Isomers

**DOI:** 10.3390/molecules28073226

**Published:** 2023-04-04

**Authors:** Silvia Alessandrini, Mattia Melosso, Víctor M. Rivilla, Luca Bizzocchi, Cristina Puzzarini

**Affiliations:** 1 Dipartimento di Chimica “Giacomo Ciamician”, Università di Bologna, Via F. Selmi 2, 40126 Bologna, Italy; 2 Centro de Astrobiología (CSIC-INTA), Ctra. de Ajalvir km. 4, Torrejón de Ardoz, 28850 Madrid, Spain

**Keywords:** astrochemistry, energetics, C_3_H_3_NO isomers, computational protocol, spectroscopic characterization

## Abstract

The C_3_H_3_NO family of isomers is relevant in astrochemistry, even though its members are still elusive in the interstellar medium. To identify the best candidate for astronomical detection within this family, we developed a new computational protocol based on the minimum-energy principle. This approach aims to identify the most stable isomer of the family and consists of three steps. The first step is an extensive investigation that characterizes the vast number of compounds having the C_3_H_3_NO chemical formula, employing density functional theory for this purpose. The second step is an energy refinement, which is used to select isomers and relies on coupled cluster theory. The last step is a structural improvement with a final energy refinement that provides improved energies and a large set of accurate spectroscopic parameters for all isomers lying within 30 kJ mol^−1^ above the most stable one. According to this protocol, vinylisocyanate is the most stable isomer, followed by oxazole, which is about 5 kJ mol^−1^ higher in energy. The other stable species are pyruvonitrile, cyanoacetaldehyde, and cyanovinylalcohol. For all of these species, new computed rotational and vibrational spectroscopic data are reported, which complement those already available in the literature or fill current gaps.

## 1. Introduction

The molecular species observed so far in the interstellar medium (ISM) are generally small and light. In fact, based on the 2021 census of the interstellar and circumstellar molecules [1], and excluding the fullerene species C_60_, C_60_^+^, and C_70_, the average number of atoms contained in the molecules on this list ranges between 5 and 6. However, it has to be noted that a relevant number of discoveries have occurred since then and updated lists can be found in the Cologne Database for Molecular Spectroscopy [2] or on the Astrochymist website [3]. Despite the limited number of atoms and the severe constraints on chemical reactivity imposed by the ISM conditions (i.e., very low temperature and density, and ionizing radiation), interstellar molecules exhibit a large variety of functional groups, including nitriles [4,5,6], imines [7,8,9,10,11,12,13], amines [14,15], alcohols [16,17,18], amides [19,20,21,22,23], aldehydes [24,25,26,27], and many others. Indeed, several chemical linkages are possible within a specific chemical formula so that a generic isomeric family can be constituted by tens—if not hundreds—of structural isomers, each characterized by different functional groups. Therefore, the recognition of the specific members of an isomeric family that are found, or can be found, in the ISM is typically a non-trivial task.

In this respect, the so-called Minimum Energy Principle (MEP) [28] offers a powerful tool. It states that (*i*) the greater the thermochemical stability of a structural isomer, the higher the expected abundance in the ISM, and (*ii*) the ratio between the abundances of two isomers is governed by their relative stabilities. With very few exceptions, such as propadienone (H_2_CCCO) [29] and acetic acid (CH_3_COOH) [28], the MEP holds. It also represents a useful guide for selecting molecular species to study in the laboratory and subsequently hunt for in the ISM; it is a solid basis for rationalizing the observed molecular abundances.

When this principle was first formulated, the question of relative stability was addressed by employing quantum-chemical calculations rooted in density functional theory (DFT), using the B3LYP method in conjunction with a basis set of triple-ζ quality to compute both the electronic energy and the zero-point vibrational energy (ZPE) contribution [30]. The rationale behind this choice was the capability of this level of theory to provide accurate molecular structures and energies, and the fact that a comparison with higher-level coupled-cluster calculations for a selection of 14 isomeric families showed overall agreement between the two sets of results. However, according to Reference [31], the uncertainty affecting B3LYP energetics can be as large as 15.5 kJ mol^−1^ when the (aug)-def2-QZVP basis set is employed. This value is two times larger than that expected in Reference [28], where a smaller basis was employed. Therefore, the B3LYP method is only appropriate when the energy difference among the isomers is quite large. This difference is likely more pronounced for smaller species and decreases as the molecular size increases, as more (and more similar) ways to link the atoms within the molecule become possible. Thus, medium-sized species require accurate methodologies for predicting the correct relative stability among structural isomers, as the energy difference between them can be as small as a few kJ mol^−1^. Moreover, the evaluation of the ZPE correction can be as important as the electronic energy, and its contribution may be decisive for the identification of the most stable isomeric [32] or conformational [33] form. Therefore, it is evident that a more stringent protocol needs to be formulated for the precise identification of the stability order within a specific isomeric family. Furthermore, the limited sizes of interstellar molecules allow pushing the computational effort to the limit.

In this work, we introduce a multi-step approach for the identification of suitable candidates, within a given isomeric family, for astronomical investigations. This protocol exploits feasible yet accurate calculations for the preliminary search of all possible isomers (and their conformers) and then proceeds to refine their energetics using a higher level of theory, thereby aiming at the selection of the most stable species. For these, even higher-level computations are then performed to further improve the structural determination as well as the energetics, with the final aim of identifying and characterizing the best candidate for astronomical searches. As a test case, we present the study of the C_3_H_3_NO isomeric family, which is thus far elusive to astronomical observations, with the aim of identifying which isomer has the highest chance of being detected in the ISM.

Astronomical observations require accurate knowledge of the rotational spectra of the species under investigation. To date, spectroscopic studies have been reported for cyanoacetaldehyde [34], cyanooxirane [35], isoxazole [36,37,38], oxazole [36,39,40], pyruvonitrile [41], and vinylisocyanate [42,43,44,45]. While measurements of the rotational spectra have been extended up to the submillimeter-wave region for some of these species [34,35,41,45], experimental data are limited to the microwave domain (i.e., below 40 GHz) for the aromatic rings, i.e., oxazole and isoxazole, and are even totally missing for other species, such as cyanovinylalcohol. Therefore, in addition to evaluating the energetics of these isomers, we provide accurate estimates of rotational, centrifugal distortion, and nuclear quadrupole coupling constants for the most interesting species. Furthermore, we review the current state of available spectroscopic information to identify possible data gaps that might prevent the detection of C_3_H_3_NO species in interstellar space.

The manuscript is organized as follows. In the following section, we present and discuss the results, starting with the preliminary energetic investigation of the C_3_H_3_NO family. Next, we report the outcomes of the second step of the protocol, which leads to the selection of the isomers for further consideration. Subsequently, we focus on the third step, which involves further structural determinations and improvements in the energetics. Section 2 concludes with the spectroscopic characterization of the most promising candidates for astronomical investigations. All details of the multi-step protocol introduced here are provided in Section 3 (Materials and Methods). Finally, the concluding remarks are reported.

## 2. Results and Discussion

In this section, the results obtained for the C_3_H_3_NO family of isomers by exploiting the multi-step protocol mentioned in the Introduction (for details, see Section 3) are presented and discussed. Although the family of C_3_H_3_NO isomers has already been studied in previous works [46,47], an exhaustive characterization of the entire isomeric family is still missing. Therefore, a systematic search of all possible isomeric forms was carried out using the SciFinder-*n* application. Bicyclic or carbene-like structures and species with separation of charge were filtered out, as our goal was to identify the most stable isomers. The numbering used in the following is based on the initial discovery of the isomers and does not follow any energetic scale.

### 2.1. Step 1: Preliminary Investigation

**Figure 1:**
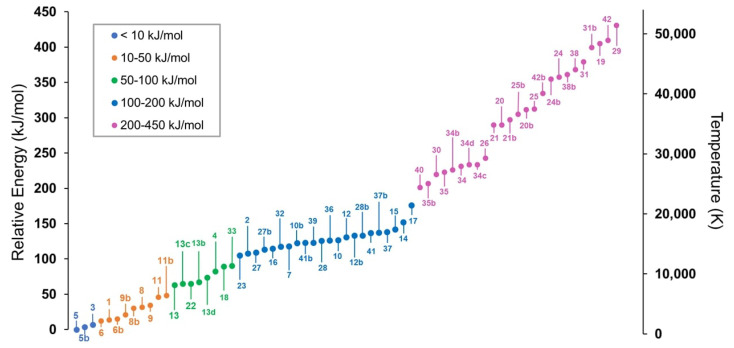
Relative energies of the 67 isomers of the C_3_H_3_NO family as obtained from the revDSD/junTZ level of theory, including the hZPE contribution. The reader may refer to the SM for the correspondence between the labeling and the molecular structure.

The starting point of our selection procedure consisted of 42 species, including the *E* and *Z* forms of structural isomers whenever applicable. In addition, other stable conformers were manually generated, resulting in a total of 67 species considered in this preliminary step. For all of them, the structure was optimized using the double-hybrid rev-DSD-PBEP86 functional [48] in conjunction with the jun-cc-pVTZ basis set [49,50], also including empirical dispersion (overall, this level is briefly denoted as revDSD/junTZ). For further details, please refer to Section 3. The revDSD/junTZ electronic energy, corrected for the harmonic ZPE (hZPE) at the same level of theory (obtained straightforwardly from harmonic force field, HFF, calculations), was used to derive the stability order of the C_3_H_3_NO isomers. The list, in decreasing order of stability, is presented in Figure 1, where the arbitrary numbering mentioned above identifies the isomers. The correspondence between the structure and label, together with revDSD/junTZ and revDSD/junTZ + hZPE relative energies, is reported in the Supporting Material (SM).

The most stable isomer at the revDSD/junTZ + hZPE level is the compound **5**, i.e., *trans*-vinylisocyanate, which thus represents our energy reference. The latter is followed by its *gauche* conformer (**5b**) and pyruvonitrile (**3**), located 3.74 and 6.75 kJ mol^−1^ higher in energy than **5**, respectively. These are the only isomers that lie below the relative energy threshold of 10 kJ mol^−1^.

The 10–50 kJ mol^−1^ energy range, represented by orange dots in Figure 1, is dominated by four isomers: cyanoacetaldehyde, oxazole, 3-hydroxy-2-propenenitrile, and 2-hydroxy-2-propenenitrile. The *trans* form of cyanoacetaldehyde (**6**) is located at 12.4 kJ mol^−1^, while its *gauche* form **6b** is about 3 kJ mol^−1^ higher in energy, i.e., at 15.8 kJ mol^−1^. The relative energy of oxazole, labeled as **1**, is 13.6 kJ mol^−1^, thus lying in between the two previously mentioned species. Moreover, 3-hydroxy-2-propenenitrile, also known as cyanovinylalcohol, presents both *Z* and *E* configurations, which in turn can show the *trans* (*t*) and *gauche* (*g*) forms, depending on the orientation of the -OH moiety with respect to the CC double bond. The revDSD/junTZ + hZPE energy scale predicts *Zg*-cyanovinylalcohol (**9b**) lying at 21.0 kJ mol^−1^, and followed by the *Eg* form (**8b**) at 30.3 kJ mol^−1^. The *Et* form of cyanovinylalcohol (**8**) is about 1 kJ mol^−1^ higher in energy than the *Eg* species, while the *Zt* conformer is the highest in energy due to steric effects and it is located at 34.5 kJ mol^−1^. Two conformers are also present for 2-hydroxy-2-propenenitrile (1-cyanoethanol); they are located at 46.3 kJ mol^−1^ (*g* form, **11**) and 48.1 kJ mol^−1^ (*t* form, **11b**).

Moving to higher energies, the four conformers of 3-iminopropenone (**13**, **13b**, **13c**, and **13d**) are located between 50 and 100 kJ mol^−1^, which is the range represented by the green dots in Figure 1. These four species differ for (i) the orientation of the imino group, with respect to the cumulenic C=C=O moiety, and (ii) the orientation of the H atom with respect to the C=N bond. The 50–100 kJ mol^−1^ range also contains the most stable isocyanide compound of the C_3_H_3_NO family, i.e., acetyl isocyanide (**22**), lying at 65.1 kJ mol^−1^, thus about 58 kJ mol^−1^ higher in energy than its cyanide counterpart (**3**). Propiolamide (**4**) is located at 82 kJ mol^−1^ and is followed by the 4-member cycle 2(3*H*)-azetone (**18**) and 3-amino-1,2-propadienone (**33**), which lie at 89.5 kJ mol^−1^ and 90.3 kJ mol^−1^, respectively.

The light-blue dots of Figure 1 represent the 100–200 kJ mol^−1^ energy range. The first member of the C_3_H_3_NO family belonging to this group, i.e., N-ethenylideneformamide (**23**), is found at 104.8 kJ mol^−1^ and it is followed by isoxazole (**2**), which lies only 3 kJ mol^−1^ above **23**. Other members of the group are (i) the isocyanic counterparts of the species mentioned before, such as isocyanoacetaldehyde (**27**), isocyanovinylalcohol (**41**, and **37**, which are the *Z* and *E* forms, respectively), and 1-isocyanoethenol (**12**); (ii) 3- or 4-member cycles. In the latter group, the main component is cyanooxirane (**7**), located at 118.0 kJ mol^−1^, which is preceded in stability by 2(1*H*)-azetone (**16**). The last species that is worth mentioning is vinylcyanide (**10**), which is the OCN counterpart of **5**.

Finally, the 200–450 kJ mol^−1^ energy range is considered. This includes 25 structures, which are represented by pink dots in Figure 1. Most of the species of this group (∼60 %) are characterized by a NO bond in the N=O or N−OH form. This suggests that nitroso and N-hydroxylamine groups are unlikely to be formed in astronomical environments. Indeed, the MEP suggests that either their formation is not favored or they exhibit an extremely low abundance of such species.

Seeking the most stable isomer within the C_3_H_3_NO family, it is necessary to improve the estimates obtained in the first step. From a computational perspective, this involves improving their electronic description and, consequently, the quantum-chemical methodology used to compute their energy. Since such an improvement can be computationally expensive, only the isomers predicted to lie below 100 kJ mol^−1^ were selected for additional computations, resulting in a total of 20 isomers.

### 2.2. Step 2: Energy Refinement

**Figure 2:**
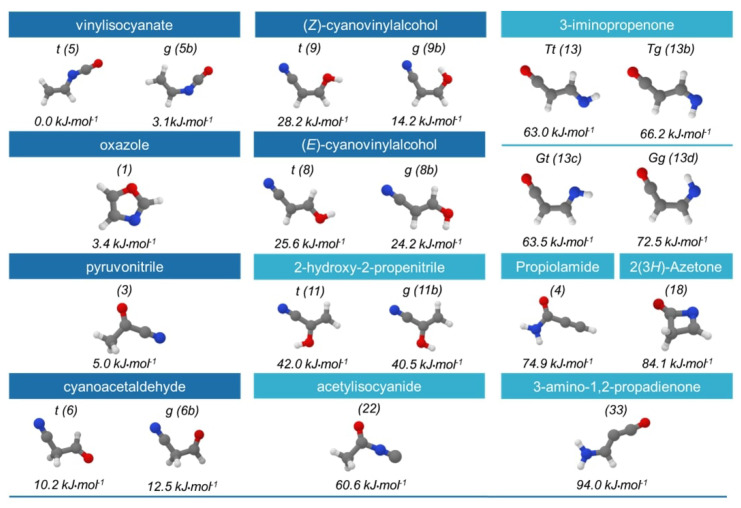
Schematic representation of the 20 isomers considered in the second step of our procedure (energy refinement). The relative energies are at the CBS + CV + hZPE level and computed on top of the revDSD/junTZ geometries. The 10 most stable isomers are reported in blue, while light blue is employed for the remaining species.

In this step, the energy of the selected 20 isomers is computed using a composite scheme based on the CCSD(T) method (coupled cluster singles and doubles approximation augmented by a perturbative treatment of triple excitations) [51]. This composite scheme accounts for extrapolation to the complete basis set (CBS) limit and the core-valence (CV) correlation effects. While the details of this composite scheme are provided in Section 3, it is abbreviated as CBS + CV hereafter. These energy computations were performed on top of the revDSD/junTZ optimized geometries, and always included the hZPE contribution at the same level of theory. A summary of the results is presented in Figure 2, which shows the CBS + CV + hZPE energies and the corresponding isomer labels.

The CBS + CV + hZPE approach still predicts *t*-vinylisocyanate as the most stable species, which is followed by its *g* conformer at 3.1 kJ mol^−1^ and then by oxazole at 3.4 kJ mol^−1^. Such a small energy difference between oxazole and *t*-vinylisocyanate compared to the revDSD/junTZ result, which is about 14 kJ mol^−1^, is remarkable. This different behavior has to be ascribed to the CBS + CV electronic energy, which predicts oxazole as the most stable isomer of the C_3_H_3_NO family, while the incorporation of the hZPE inverts the trend between the latter and *t*-vinylisocyanate. Moving to higher energies, oxazole is followed by pyruvonitrile (**3**), at 5.0 kJ mol^−1^, and then by cyanoacetaldehyde (**6**), at 10.2 kJ mol^−1^. The remaining isomers exhibit the same energetic order as that obtained from the revDSD/junTZ calculations. Overall, the CBS + CV energies differ in absolute terms from the revDSD counterparts by an average of 4.2 kJ mol^−1^, with the largest discrepancy observed for oxazole, which is predicted to be 10 kJ mol^−1^ lower in energy by CBS + CV + hZPE.

**Figure 3:**
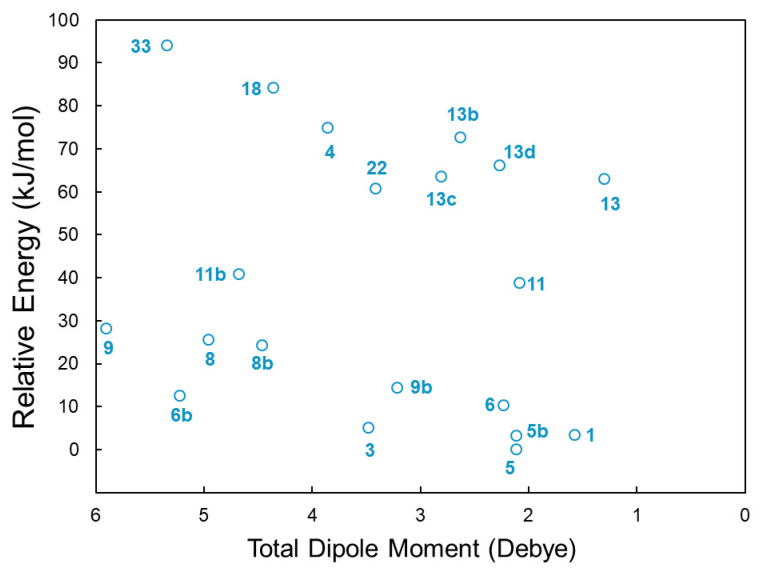
Total electric dipole moment (revDSD/junTZ) plotted against relative energy (CBS + CV + hZPE) for the 20 most stable isomers of the C_3_H_3_NO family. For labels, see Figure 2 and the text.

Useful information on potential candidates for radioastronomical detection is already obtained in this step of the protocol. The possibility of observing a given molecule in the ISM depends on the intensity of its rotational transitions. While the abundance of the molecule in the environment under consideration clearly plays a role, the rotational line intensity is proportional to the square of the electric dipole moment. Therefore, the value of the latter needs to be considered together with relative energy. For this reason, Figure 3 plots the total electric dipole moment for the 20 most stable isomers, computed at the revDSD/junTZ level, against their relative CBS + CV + hZPE energy. This figure points out that the most stable species of the family (namely, **5**, **5b**, and **1**) have a small dipole moment in comparison with the other members. All forms of cyanovinylalcohol are favored by the large dipole moment, but pyruvonitrile (**3**) and the *trans* form of cyanoacetaldehyde (**6b**) seem to be those that better combine a low relative energy and a large dipole moment. The other species are very high in energy, and this is not counterbalanced by the magnitude of the dipole moment, making their detection unlikely. Interestingly, the gas-phase formation of cyanoacetaldehyde has been suggested as feasible in the ISM via the reaction between the CN radical and oxirane [52], both species being already detected in different interstellar regions (see Reference [52] for details). The same consideration applies to cyanovinylalcohol, which can be easily formed from the reaction between the CN radical and vinylalcohol, the latter being present in the ISM as well [18,53,54]. Pyruvonitrle is instead a co-product of the reaction between acetahldehyde and the CN radical [55].

The number of species of the C_3_H_3_NO family that are potentially observable in the ISM is narrowed by employing the more accurate energy list of Figure 2 and the dipole moment considerations from Figure 3. Therefore, only the species within 30 kJ mol^−1^, with respect to the most stable species (**5**), which are 10 isomers, have been selected for the next step of the procedure, i.e., the structural improvement.

### 2.3. Step 3: Structure Improvement and Final Energy Refinement

In this step, the molecular structures of the 10 selected isomers (blue-colored molecules in Figure 2) were refined to further improve the electronic energies and provide accurate estimates of the corresponding rotational parameters, especially for those molecules that could potentially be present in the ISM and have not been studied experimentally via rotational spectroscopy. To achieve this goal, the CBS + CV composite scheme is employed in geometry optimizations, yielding improved equilibrium rotational constants (which only depend on the molecular equilibrium structure) and electronic energies (obtained on top of geometries at the same level of theory). To predict the rotational constants accurately, the vibrational corrections to the equilibrium counterparts have to be computed, requiring anharmonic force field calculations that have been performed at the revDSD/junTZ level of theory (see Section 3 for details). These anharmonic computations also provide better estimates of the ZPE corrections (anharmonic ZPE, aZPE), which further improve the relative energy scale of isomer stability. While this section discusses the new energetic trend, the spectroscopic characterization is addressed in the next section.

Moving on from the CBS + CV + hZPE level (revDSD/junTZ structure) to the CBS + CV + aZPE one (CBS + CV geometry; see Table S2), the relative energy of each isomer remains nearly unchanged; this means that—as expected [56,57]—double-hybrid DFT geometries are suitable for high-level energy computations and the anharmonic contribution to the ZPE correction is small. Indeed, the latter accounts—on average—for less then 2 kJ mol^−1^. Oxazole is now predicted as the second most stable species, followed by the *g*-vinylisocyanate, which are at 3.2 kJ mol^−1^ and 3.3 kJ mol^−1^, respectively, above *t*-vinylisocyanate.

Since the last step of our computational protocol leads to oxazole and *t*-vinylisocyanate as the two most stable isomers lying close in energy, a final refinement is required to confirm (or not) the latter species as the most stable form of the C_3_H_3_NO family. For this purpose, the electronic energy was computed using the HEAT-like approach [58,59], which incorporates higher-order terms in the coupled cluster expansion and relativistic effects (see Section 3); moreover, the aZPE correction was improved by means of a hybrid approach, which combines the HFF at the fc-CCSD(T)/cc-pVTZ level with the revDSD/junTZ anharmonic contribution. At the CBS + CV/CBS + CV level (which means that the CBS + CV level has been used for both structure and energy), oxazole is located −4.52 kJ mol^−1^ below *t*-vinylisocyanate. The incorporation of the fT contribution (i.e., the full treatment of triple excitations) reduces the energy difference to −2.86 kJ mol^−1^, thus favoring *t*-vinylisocyanate by 1.66 kJ mol^−1^. The effect of the perturbative treatment of quadruple excitations (pQ) is counterbalanced by the DBOC term (which is the diagonal Born–Oppenheimer correction, see Section 3), thus confirming −2.86 kJ mol^−1^ as the energy gap. The scalar relativistic contribution further reduces the energy difference to −2.73 kJ mol^−1^. Overall, the HEAT-like approach predicts oxazole as the most stable isomer when considering only the electronic energy. However, the ZPE term has to be taken into account. Its incorporation as hZPE at the fc-CCSD(T)/cc-pVTZ level favors the *trans* form of vinylisocyanate by 8.4 kJ mol^−1^, thus locating oxazole at 5.6 kJ mol^−1^ above the former. The anharmonic correction to the hZPE contribution has a small impact on the overall value, leading to an energy difference of 5.4 kJ mol^−1^ and establishing *t*-vinylisocyanate as the most stable isomer of the C_3_H_3_NO family.

The energy gap between oxazole and *t*-vinylisocyanate is strongly affected by the vibrational energy, as observed for the two conformers of cyclopropanecarboxaldehyde [33]; this can be attributed to the high vibrational frequencies of oxazole, a stable aromatic species, in contrast to the flexible *t*-vinylisocyanate.

### 2.4. Spectroscopic Characterization

Several C_3_H_3_NO isomers have already been characterized by means of rotational spectroscopy. Rotational transitions have been reported for high-energy isomers, such as propiolamide (**4**) [60] and isoxazole (**2**) [36,37,38]. Similarly, six isomers among the ten most stables species (according to the present protocol), have also been experimentally observed. These are conformers of (*i*) vinylisocyanate [42,43,44], (*ii*) cyanoacetaldehyde [34], (*iii*) pyruvonitrile [41], and (*iv*) oxazole [36,39,40,61]. The only species that have not yet been experimentally characterized are the four conformers of cyanovinylalcohol, which are the remaining molecules in the top 10 of the stable isomers. Therefore, our protocol has been employed to derive accurate rotational spectroscopic parameters of the six isomers that have been experimentally studied, which can be used to estimate the uncertainties affecting our predictions for cyanovinylalcohol.

Table 1 reports the CBS + CV equilibrium rotational constants, corrected for vibrational contributions at the revDSD/junTZ level, for the species that have already been experimentally characterized. The $A_{0}$ rotational constant is reproduced with a relative error of 0.5%, while $B_{0}$ and $C_{0}$ have both a mean error of 0.1%. The average error on $A_{0}$ significantly reduces to 0.3% if one removes from the statistics the $A_{0}$ value for *t*-NCCH_2_CHO, which might be affected by experimental misassignments, as suggested in Reference [34]. These conservative estimates for uncertainty can be applied to the rotational constants obtained for the four isomers of cyanovinylalcohol collected in Table 2, where the centrifugal distortion and nuclear quadrupole coupling constants, together with the electric dipole moment components, are also reported.

The values of Table 2 can be compared with those of Reference [34], where the conformers of cyanovinylalcohol were investigated by means of the second-order Møller–Plesset perturbation theory (MP2) in conjunction with the aug-cc-pVTZ basis set. The energy differences reported in Reference [34] are consistent with our computations, which, however, predict that all conformers are planar and reveal the presence of a *trans* form for (*E*)-cyanovinylalcohol that was not reported in the previous study. The rotational constants predicted in Reference [34] refer to the equilibrium structure and exhibit deviations with respect to ours up to 100 MHz ($A_{0}$, (*E*)-*g*-cyanovinylalcohol). In most cases, the discrepancies are larger than the vibrational corrections, which should be included whenever aiming for accurate estimates [62,63], thus highlighting the inadequacy of the MP2 method in predicting rotational constants of spectroscopic accuracy. There is no comparison available for centrifugal distortion constants and nuclear quadrupole coupling constants, which are also essential parameters to ensure a correct assignment of rotational transitions.

**Figure 4:**
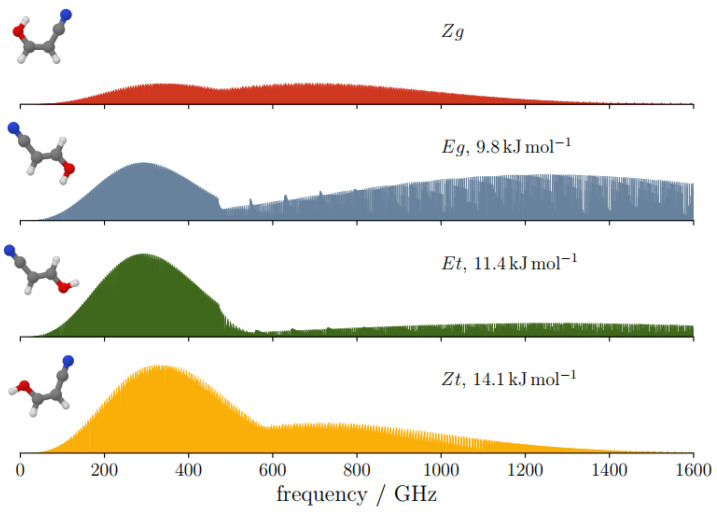
Simulated rotational spectra of the four isomers of cyanovinylalcohol based on the computed constants reported in Table 2. The calculations were performed for *J* ranging in the 0–100 interval and T=300 K. The *y*-axes are normalized with respect to the most intense Zt spectrum. The relative energy at the CBS + CV + aZPE level of theory is provided with respect to the most stable isomer (Zg).

The spectroscopic constants of the four forms of cyanovinylalcohol were used to simulate their rotational spectra, which are reported in Figure 4. The intensity of the transitions follows the opposite trend of stability, i.e., the most intense spectrum is observed for the less stable species. However, at room temperature, all of the spectra show a peak of intensity at around 300 GHz; this will be helpful for future laboratory studies.

For the 10 most stable isomers of the C_3_H_3_NO family, our computational methodology allowed for the accurate determination of anharmonic vibrational frequencies in addition to the rotational parameters, which can be useful in guiding the assignment of high-resolution infrared (IR) spectra. Table 3 reports our best anharmonic values for oxazole and *t*-vinylisocyanate, obtained with a hybrid approach that combines fc-CCSD(T)/cc-pVTZ harmonic frequencies with anharmonic revDSD/junTZ contributions. The computed anharmonic vibrational frequencies for oxazole reproduce experimental data with errors below 5 cm^−1^ in the 600–1400 cm^−1^ range, as evident from the comparison with available experimental data. For *t*-vinylisocyanate, vibrational frequencies have not been measured experimentally, and thus our values represent the most accurate data available in the literature.

For the other eight species lying below 30 kJ mol^−1^, the anharmonic revDSD/junTZ vibrational frequencies are reported in the SM. In this case, the comparison with experimental data can be performed only for pyruvonitrile. For this isomer, it is noted that the assignment of the band observed at 535 cm^−1^ to the $\nu_{16}$ fundamental [64] casts some doubts. In fact, for the same mode, our calculations predict a value of 584.86 cm^−1^ and, thus, 50 cm^−1^ above the observed one. However, such a deviation is five times larger than the average deviation observed for the other fundamental bands.

## 3. Materials and Methods

**Figure 5:**
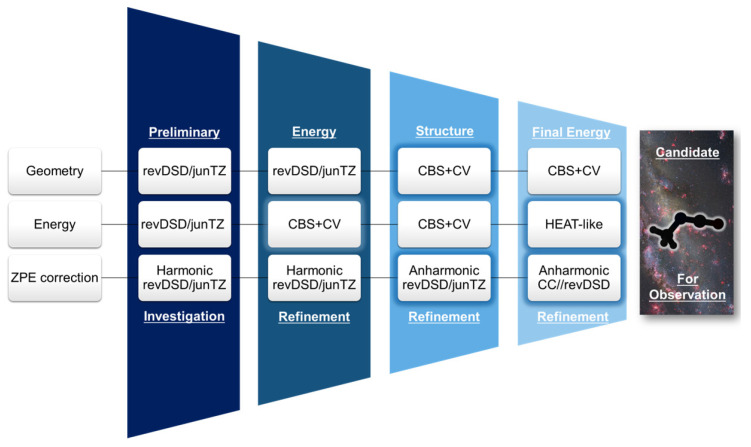
Schematic representation of the computational protocol developed in this work for the individuation of potential candidates for astronomical observations.

In this work, a three-step protocol based on the MEP was introduced and exploited to identify potential candidates for astronomical detection among the isomers of the C_3_H_3_NO family. The procedure is schematized in Figure 5 and explained in detail in the subsequent sections. All DFT calculations were performed using the Gaussian16 software suite [65], while the CFour program package [66,67] and MRCC [68] (interfaced with CFour) were used for methodologies based on the coupled cluster (CC) approach [69].

### 3.1. Step 1: Preliminary Investigation

The first step of the procedure is the preliminary investigation of a large number of molecules having the same molecular formula, i.e., C_3_H_3_NO in this case. This step involves a large number of compounds and needs to be carried out using an affordable computational method that can provide reliable data. For this reason, as mentioned in Section 2, all species of the C_3_H_3_NO family were analyzed at the revDSD/junTZ level. All DFT computations were corrected for Grimme’s D3 empirical dispersion, including the BJ dumping function [70,71]. The jun-cc-pVTZ set is a partially augmented triple-zeta basis set that balances computational cost and the inclusion of diffuse functions. For each isomer, the revDSD/junTZ level of theory was used to obtain the equilibrium molecular structure, the corresponding electronic energy, and the analytic harmonic force field. The force field was used to ensure the nature of the stationary point and to provide the harmonic zero-point energy correction (hZPE) to the electronic energy.

The selection of initial structures to be considered is left to the user, but in this case, Sci-Finder was employed to obtain a large number of initial structures. An automatic procedure based on genetic algorithms guiding semi-empirical methods [72,73,74] is currently under development.

### 3.2. Step 2: Energy Refinement

For the isomers within a specific energy range (for example, 0–100 kJ mol^−1^, based on the preliminary investigation at step 1), the electronic energy is further improved by exploiting a composite scheme rooted in the CC theory on top of the revDSD/junTZ geometries. By definition, composite schemes include different contributions, each of them computed at the best possible level of theory according to the size of the system, in order to minimize errors associated with quantum-chemical calculations. In view of the number of isomers considered and their sizes, the present protocol uses the CBS + CV approach [33,56,75,76,77,78] for the electronic energy (E(CBS+CV)):

(1) $$E\left(\mathrm{CBS}+\mathrm{CV}\right)=E_{\mathrm{HF}-\mathrm{SCF}}^{\infty }+E_{\mathrm{CCSD}(T)}^{\infty }+\Delta E\left(\mathrm{CV}\right)$$

The first term on the right-hand side of Equation (1) is the extrapolation to the CBS limit of the Hartree–Fock self-consistent field (HF-SCF) energy, which is evaluated with the exponential formula by Feller [79], requiring three energy computations. These are carried out with the cc-pV*n*Z family of basis sets, using n= T, Q, and 5 [49]. The second contribution in Equation (1) accounts for the CBS limit of the CCSD(T) correlation energy, which is estimated via the two-point $n^{-3}$ formula [80], using the cc-pVTZ and cc-pVQZ basis sets. Since these computations are carried out within the frozen-core (fc) approximation, the last term on the right-hand side of Equation (1) incorporates the effects of correlating the inner-shell electrons, which is the CV correlation term. The ΔE(CV) is computed as the energy difference between all-electron (ae) and fc calculations, both carried out with the same basis set, cc-pCVTZ [81]. The CBS + CV electronic energies augmented for hZPE were then employed for a new classification of the C_3_H_3_NO isomers, and only those in the 0–30 kJ mol^−1^ energy range were retained in the third step.

### 3.3. Step 3: Structure Improvement & Final Energy Refinement

The third step of our computational protocol is used to obtain improved structural determinations with the aim of further improving the electronic energies, as well as laying the basis for an accurate spectroscopic characterization. For this purpose, the CBS + CV energy of Equation (1) is used to build an energy gradient (dE(CBS+CV)/dx), which is minimized to obtain the corresponding equilibrium geometry [82,83]:

(2) $$\frac{dE_{\left(\mathrm{CBS}+\mathrm{CV}\right)}}{dx}=\frac{dE^{\infty }\left(\mathrm{HF}-\mathrm{SCF}\right)}{dx}+\frac{d\Delta E^{\infty }\left(\mathrm{CCSD}(T)\right)}{dx}+\frac{d\Delta E\left(\mathrm{CV}\right)}{dx}\;.$$

In addition to the CBS + CV equilibrium structures, the above scheme provides CBS + CV electronic energies on top of the geometries optimized at the same level of theory. These energies, augmented for aZPE (CBS + CV + aZPE), are used to determine the final stability order of the C_3_H_3_NO isomers considered in step 3.

Specifically, this step was applied to the 10 isomers lying within 30 kJ mol^−1^ above the most stable species. The accurate CBS + CV equilibrium structures of these isomers straightforwardly provide their equilibrium rotational constants ($B_{e}$). Using the vibrational perturbation theory to the second order (VPT2) [84], the rotational constants of the vibrational ground state consist of two terms:

(3) $$B_{0}^{\gamma }=B_{e}^{\gamma }+\Delta B_{0}^{\gamma }\;,$$

where $B_{0}^{\gamma }=A_{0},\;B_{0},\;$ and $C_{0}$ for γ=a,b, and *c*, respectively. As mentioned above, the $B_{e}^{\gamma }$ term depends on the equilibrium molecular structure, but also on its atomic composition. The vibrational correction ($\Delta B_{0}$) is obtained as the half-sum over the normal modes (*r*) of the vibration–rotation interaction constants ($\alpha_{r}^{\gamma }$). [84]:

(4) $$\Delta B_{0}^{\gamma }=-\frac{1}{2}\sum_{r}\alpha_{r}^{\gamma }\;.$$

The $B_{e}^{\gamma }$ value accounts for about 99% of $B_{0}^{\gamma }$ and, thus, strongly affects its accuracy [62,85]. Therefore, the equilibrium geometries of the species of interest have been evaluated at the CBS + CV level to ensure the accuracy of the $B_{0}$ value. To calculate the $\Delta B_{0}$ term, an anharmonic force field is required. Although its contribution is small, the vibrational correction is evaluated at the revDSD/junTZ level due to its affordability. Additionally, these anharmonic computations provide other spectroscopic parameters for rotational spectroscopy such as the centrifugal distortion constants up to sextic terms, electric dipole moment, and nuclear quadrupole coupling constants, along with anharmonic vibrational frequencies and the corresponding ZPE contribution (aZPE) [86].

The final energy refinement is part of the third step of the protocol, but it is not mandatory. Instead, it should be employed whenever two or more relevant isomers are particularly close in energy so that a sub-kJ mol^−1^ accuracy is required to assure the relative energy. In this case, the electronic energy at the CBS + CV level (E(CBS+CV)) is augmented by higher-order terms to exploit the HEAT-like protocol (*E*(HEAT-like)) [58,59]:

(5) E(HEAT-like)=E(CBS+CV)+ΔE(fT)+ΔE(pQ)+ΔE(DBOC)+ΔE(rel).

The second term on the right-hand side of Equation (5) represents the contribution from the full treatment of triple excitations, which is computed as the energy difference between CCSDT [87,88] and CCSD(T) energies using the cc-pVTZ basis set. Here, CCSDT denotes the CC singles, doubles, and triples method. The term ΔE(pQ) takes into account the effect of quadruple excitations and is computed using the CCSDT(Q) method [89,90,91] (which is CCSDT augmented by a perturbative treatment of quadruples), employing the cc-pVDZ basis set. The remaining two terms account for the diagonal Born–Oppenheimer correction (DBOC), computed at the HF-SCF level using the aug-cc-pVTZ basis set [92], and the scalar relativistic term, computed at the CCSD(T) level in conjunction with the aug-cc-pCVTZ basis set. This last term includes only one-electron mass-velocity and Darwin corrections and is obtained using perturbative techniques [93,94].

The E(HEAT-like) energy needs to be corrected for the ZPE term, which is purposely improved by using the harmonic fc-CCSD(T)/cc-pVTZ data and the anharmonic contribution at the revDSD/junTZ level. This hybrid anharmonic force field has already been used in Reference [33] and allows retrieving accurate vibrational frequencies of the species considered in the final refinement.

## 4. Conclusions

The computational protocol developed in this study provides a systematic and simple methodology to investigate families of isomers of astrochemical interest using the MEP as a guiding reference.

The first step of the procedure involves a preliminary investigation of all possible isomers and conformers, which were 67 in the case of the C_3_H_3_NO family. This step is carried out using a double-hybrid DFT functional in conjunction with a partially augmented triple-zeta basis set, which is able to provide a reliable energy "ladder" when the hZPE correction is included. The results from this step are used to select a smaller group of species, for example, those within 100 kJ mol^−1^ above the most stable isomer, which are retained for the next step. In the second step, a more accurate computational methodology is used to improve the electronic energy of the selected isomers. To accomplish this, a composite scheme rooted in the CC theory, the CBS + CV approach, is used to compute the electronic energy while retaining the double-hybrid reference geometries and the corresponding hZPE. Moving from the first to the second step, the relative energy can change up to 10 kJ mol^−1^, as noted for oxazole, and several changes in the relative energy scale might occur. The third step of the protocol involves a limited number of species, i.e., those lying below 30 kJ mol^−1^, according to the results of the previous step. At this stage, the aim is to further improve the energetics while providing an accurate set of rotational and vibrational spectroscopic data. This is accomplished by optimizing the molecular structures at the CBS + CV level and extending the determination of the force field by incorporating the anharmonic terms at the revDSD/junTZ level. Overall, these computations provide a comprehensive set of rotational parameters, ranging from rotational to centrifugal distortion and nuclear quadrupole coupling constants, as well as anharmonic vibrational frequencies, which are useful for interpreting IR spectra. The third step of our protocol results in only small energy changes, with oxazole becoming the second most stable isomer, situated between two vinylisocyanate conformers. To establish the most stable species with sub-kJ mol^−1^ accuracy, a non-mandatory step of the procedure, referred to as final energy refinement, is carried out using the HEAT-like composite scheme. At this level, oxazole is predicted to be the most stable species by about 3 kJ mol^−1^, but the smaller zero-point energy contribution of *t*-vinylisocyanate, in comparison to that of the aromatic ring, establishes *t*-CH_2_CHNCO as the most stable isomer within the C_3_H_3_NO family.

In conclusion, based on the MEP, the first isomer of the C_3_H_3_NO family that should be detected in the ISM is the *trans* form of vinylisocyanate. To date, this species has been searched for in the G + 0.693-0.027 molecular cloud [95] and the Sgr B2(N) region [45]. In both cases, only upper limits on its abundance could be derived, which are $2.5\times 10^{12}$ cm^−2^ and $2.4\times 10^{16}$ cm^−2^ for G + 0.693 and Sgr B2(N), respectively. To the best of our knowledge, no searches have been reported towards the cold core of the Taurus Molecular Cloud (TMC-1), one of the richest and most studied dark clouds. However, the high sensitivity of the QUIJOTE [96] and GOTHAM [97] line surveys (with a noise level below 1 mK) should allow for the identification of *trans* vinylisocyanate even if its column density is as low as ∼$10^{11}$ cm^−2^. Furthermore, our protocol indicates that cyanovinylalcohol and cyanoacetaldehyde are potential candidates for detection in the ISM. However, laboratory spectroscopic studies are still missing for cyanovinylalcohol, thus our study provides a comprehensive set of rotational and vibrational spectroscopic parameters to assist future experimental work.

## Figures and Tables

**Table 1:** Experimental ^a^ (Exp.) and computed ^b^ (Theory) rotational constants of the vibrational ground state (MHz) for vinylisocyanate (*t* and *g*), oxazole, cyanoacetaldehyde (*t* and *g*) and pyruvonitrile.

	CH_2_CHNCO				*c*-N(CH)_2_OCH	
	*trans*		*gauche*			
	Theory	Exp.	Theory	Exp.	Theory	Exp.
$A_{0}$	62,759.3 (0.28%)	62,586.3	20,306.5 (0.81%)	20,144.1	10,058.0 (0.07%)	10,051.0
$B_{0}$	2437.2 (0.02%)	2437.8	3097.7 (0.31%)	3107.5	9659.1 (0.14%)	9645.8
$C_{0}$	2346.2 (0.01%)	2346.5	2684.7 (0.17%)	2689.4	4924.1 (0.10%)	4919.4
	NCCH_2_CHO				CH_3_COCN	
	*trans*		*gauche*			
	Theory	Exp.	Theory	Exp.	Theory	Exp.
$A_{0}$	27,147.3 (1.46%)	267,56.8	12,827.5 (0.12%)	12,812.1	10,194.2 (0.08%)	10,185.9
$B_{0}$	2569.5 (0.25%)	2576.0	3657.5 (0.03%)	3658.7	4157.6 (0.004%)	4157.7
$C_{0}$	2416.9 (0.28%)	2423.6	2894.5 (0.002%)	2894.6	3003.5 (0.03%)	3002.8

^a^ See text for experimental references. ^b^ CBS + CV equilibrium rotational constants corrected for the vibrational contribution at the revDSD/junTZ level. Relative deviations from the experiment are given in parentheses.

**Table 2:** Computed rotational parameters for the (*E*)- and (*Z*)-cyanovinylalcohol forms and their relative energies.

Parameter ^a^	Units	(*Z*)-Cyanovinylalcohol		(*E*)-Cyanovinylalcohol	
		*trans*	*gauche*	*trans*	*gauche*
$A_{0}$	MHz	13,893.0	12,869.2	45,505.9	44,451.1
$B_{0}$	MHz	3554.5	3845.5	2387.0	2384.3
$C_{0}$	MHz	2827.2	2956.3	2267.2	2262.0
$D_{J}$	kHz	3.1	4.6	0.33	0.33
$D_{JK}$	kHz	−27.3	−32.6	−35.7	−32.54
$D_{K}$	kHz	117.1	100.3	2330.0	1981.70
$d_{1}$	kHz	−0.95	−1.5	−0.041	−0.040
$d_{2}$	Hz	−60.6	−0.1	−0.92	−0.96
$H_{J}$	Hz	0.011	0.025	0.00033	0.00032
$H_{JK}$	Hz	−0.072	−0.18	−0.072	−0.067
$H_{KJ}$	Hz	−0.52	−0.28	2.2	2.2
$H_{K}$	Hz	3.3	2.4	69.8	32.2
$h_{1}$	Hz	0.0054	0.012	0.0001	0.0001
$h_{2}$	mHz	0.88	1.8	0.0031	0.0031
$h_{3}$	mHz	0.16	0.35	0.0013	0.0013
$\chi_{aa}$	MHz	−2.1	−1.6	−1.3	−3.6
$\chi_{bb}$ − $\chi_{cc}$	MHz	−1.8	−1.67	0.1	0.4
$\chi_{ab}$	MHz	3.0	3.3	0.7	1.85
$\left|\mu_{a}\right|$	D	5.45	2.60	4.78	4.03
$\left|\mu_{b}\right|$	D	2.16	1.83	0.93	1.72
ΔE	kJ mol^−1^	14.1	0.0	11.4	9.8

^a^ Rotational constants of the vibrational ground state obtained from the CBS + CV equilibrium terms augmented for the vibrational correction at the revDSD/junTZ level. Centrifugal distortion and nuclear quadrupole coupling constants as well as vibrationally averaged dipole moment components from the revDSD/junTZ anharmonic force field calculations. Watson’s Hamiltonian, *S*-reduction, $III^{r}$ representation. Relative energies at the CBS + CV + aZPE level of theory.

**Table 3:** Computed vibrational frequencies of oxazole and *t*-vinylisocyanate compared to the available experimental values. All values are in cm^−1^.

	oxazole			
	CC/TZ ^a^	$\Delta \nu_{\mathrm{anh}}$ ^b^	best Estimate	Exp. ^c^
$\nu_{1}$	3309.09	−126.55	3182.54	
$\nu_{2}$	3285.40	−126.81	3158.59	
$\nu_{3}$	3275.62	−125.38	3150.24	
$\nu_{4}$	1575.76	−35.97	1539.79	
$\nu_{5}$	1540.35	−43.16	1497.19	
$\nu_{6}$	1358.40	−32.97	1325.43	1329.75165(3)
$\nu_{7}$	1280.23	−27.82	1252.41	
$\nu_{8}$	1177.36	−34.36	1143.00	1142.50528(3)
$\nu_{9}$	1112.64	−26.01	1086.63	1091.12069(5)
$\nu_{10}$	1108.71	−28.00	1080.70	1081.29060(4)
$\nu_{11}$	1074.50	−23.58	1050.92	1051.75844(3)
$\nu_{12}$	919.16	−13.30	905.86	909.28465(6)
$\nu_{13}$	908.75	−13.73	895.01	899.33009(6)
$\nu_{14}$	872.69	−18.31	854.38	859.19(1)
$\nu_{15}$	851.29	−18.58	832.71	832.01870(3)
$\nu_{16}$	763.10	−17.15	745.95	749.31060(3)
$\nu_{17}$	655.72	−11.31	644.41	646.35537(3)
$\nu_{18}$	618.04	−10.21	607.83	
	*t*-vinylisocyanate			
	CC/TZ ^a^	$\Delta \nu_{\mathrm{anh}}$ ^b^	best estimate	Lit. ^d^
$\nu_{1}$	3270.82	−137.94	3132.87	3149.9
$\nu_{2}$	3186.24	−132.33	3053.92	3079.0
$\nu_{3}$	3168.87	−106.84	3062.03	3051.5
$\nu_{4}$	2324.44	−60.72	2263.71	2301.7
$\nu_{5}$	1689.63	−53.05	1636.58	1635.6
$\nu_{6}$	1484.92	−28.65	1456.27	1490.3
$\nu_{7}$	1405.70	−33.45	1372.25	1408.3
$\nu_{8}$	1335.24	−26.77	1308.48	1328.3
$\nu_{9}$	1115.46	−22.70	1092.77	1109.3
$\nu_{10}$	972.93	−14.57	958.36	972.1
$\nu_{11}$	899.79	−3.96	895.84	915.6
$\nu_{12}$	854.82	0.76	855.58	853.5
$\nu_{13}$	694.68	1.63	696.31	698.4
$\nu_{14}$	639.59	−24.99	614.60	656.0
$\nu_{15}$	573.32	−17.43	555.89	601.1
$\nu_{16}$	446.77	−11.57	435.21	453.1
$\nu_{17}$	143.04	−2.45	140.58	136.5
$\nu_{18}$	83.22	3.45	86.67	78.3

^a^ Harmonic frequencies at the fc-CCSD(T)/cc-pVTZ level of theory. ^b^ Anharmonic corrections at the revDSD/junTZ level. ^c^ Experimental data taken from Reference [61]. ^d^ The literature values computed at the CCSD/cc-pVTZ level (see Reference [45]).

## Data Availability

The original contributions presented in the study are included in the article Supplementary Materials (for details see above), further inquiries can be directed to the corresponding author.

## Supplementary Materials

The supporting information can be downloaded at: https://www.mdpi.com/article/10.3390/molecules28073226/s1. The information includes: the list of revDSD/junTZ and revDSD/junTZ + hZPE relative energies, together with the corresponding isomeric labels and structures (Table S1). CBS + CV and CBS + CV + aZPE relative energies for the ten most stable isomers obtained on top of the CBS + CV geometries (Table S2). Optimized CBS + CV geometries for the ten most stable isomers in Cartesian coordinates (Tables S3–S7) together with the anharmonic frequencies computed at the revDSD/junTZ level for the same species. Harmonic and anharmonic ZPE corrections computed at the revDSD/junTZ level (Tables S8–S11).

## Abbreviations

The following abbreviations are used in this manuscript:

ISM	interstellar medium
MEP	minimum energy principle
DFT	density functional theory
ZPE	zero-point vibrational energy
HF-SCF	Hartree–Fock self-consistent field
CC	coupled cluster
CCSD(T)	coupled cluster with single, double, and perturbative triple excitations
CBS	complete basis set
CV	core-valence
CCSDT	coupled-cluster with single, double, and triple excitations
CCSDT(Q)	coupled-cluster single, double, triple, and perturbative quadrupole excitations
DBOC	diagonal Born–Oppenheimer correction
MP2	second-order Møller-Plesset perturbation theory
VPT2	second-order vibrational perturbation theory (VPT2)
HFF	harmonic force field
