# Supplementary material for: Computational Protocol for the Identification of Candidates for Radioastronomical Detection and Its Application to the C3H3NO Family of Isomers
Source: Molecules. 2023 Apr 4;28(7):3226. doi: 10.3390/molecules28073226 (PMC10096335; doi:10.3390/molecules28073226)
Supplement: Supplementary file 1 [file molecules-28-03226-s001.zip › molecules-2307192-supplementary.pdf]

Supporting Information for "Computational Protocol for the Identification of Candidates for Radioastronomical Detection and its Application to the C_3H_3NO Family of Isomers"

Silvia Alessandrini, Mattia Melosso, Víctor M. Rivilla,
Luca Bizzocchi, and Cristina Puzzarini

March 11, 2023

1 Energetics from the Preliminary Investigation

Table S1: revDSD/junTZ and revDSD/junTZ+hZPE relative energies for all the species considered in the preliminary investigation. For the sake of completeness, the labels employed in fig. 1 of the main text is reported together with the name and a schematic representation of the species. For vinylisocianate, the species of reference, the energy is also reported in Hartree.

TAG	Structure	Name	revDSD/junTZ kJ mol^{-1}	revDSD/junTZ + hZPE kJ mol^{-1}
5	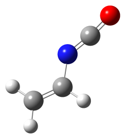	*t*-vinylisocyanate	-245.73005 Ha<br>(0.0 kJ mol^{-1})	0.05563 Ha<br>(0.0 kJ mol^{-1})
5b	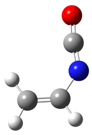	*g*-vinylisocyanate	3.42	3.74
3	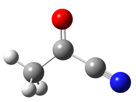	pyruvonitrile	9.91	6.75
6	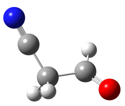	*t*-cyanoacetaldehyde	14.74	12.41

Continued on next page

TAG	Structure	Name	revDSD/junTZ kJ mol^{-1}	revDSD/junTZ +hZPE kJ mol^{-1}
1	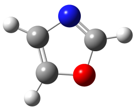	oxazole	5.61	13.59
6b	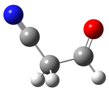	*g*-cyanoacetaldehyde	17.62	15.38
9b	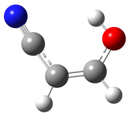	(*Z*)-*g*-cyanovinylalcohol	19.08	20.97
8b	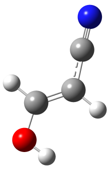	(*E*)-*g*-cyanovinylalcohol	29.34	30.24
8	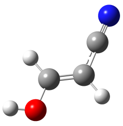	(*E*)-*t*-cyanovinylalcohol	31.55	31.81
9	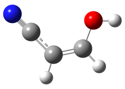	(*Z*)-*t*-cyanovinylalcohol	33.64	34.49
11	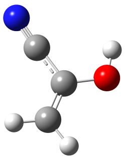	*t*-2-hydroxy-2-propenitrile	48.05	46.27
11b	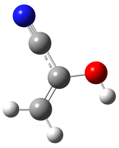	*t*-2-hydroxy-2-propenitrile	49.44	48.14

Continued on next page

TAG	Structure	Name	revDSD/junTZ kJ mol^{-1}	revDSD/junTZ +hZPE kJ mol^{-1}
13	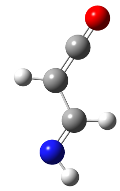	*Tt*-3-imino-1-propen-1-one	63.89	63.13
13c	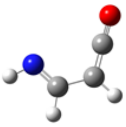	*Gt*-3-imino-1-propen-1-one	65.40	64.75
22	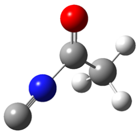	acetylisocyanide	68.91	65.07
13b	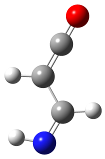	*Tg*-3-imino-1-propen-1-one	67.73	67.09
13d	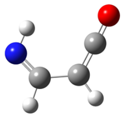	*Gg*-3-imino-1-propen-1-one	74.95	73.90
4	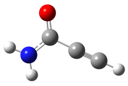	Propiolamide	85.43	82.61
18	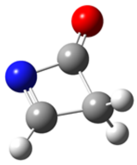	*2(3H)*-azetone	87.37	89.52
33	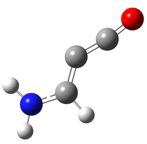	3-amino-1,2-propadien-1-one	88.59	90.28

Continued on next page

TAG	Structure	Name	revDSD/junTZ kJ mol^{-1}	revDSD/junTZ +hZPE kJ mol^{-1}
23	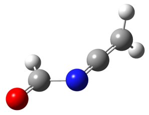	N-ethenylideneformamide	111.09	104.76
2	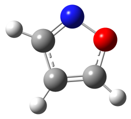	isoxazole	101.55	107.82
27	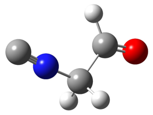	*t*-isocyanoacetaldehyde	111.96	108.76
27b	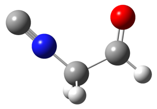	*g*-isocyanoacetaldehyde	115.73	113.17
16	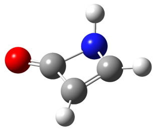	*2(1H)*-azetone	112.62	114.57
32	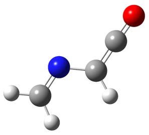	methyleneamino-ethenone	120.62	117.26
7	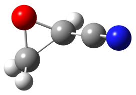	cyanooxirane	116.26	117.95
10b	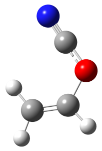	*g*-vinylcyanate	124.04	122.50
41b	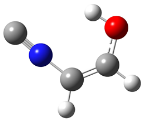	(Z)-*g*-isocyanovinylalcohol	122.12	122.65

Continued on next page

TAG	Structure	Name	revDSD/junTZ kJ mol^{-1}	revDSD/junTZ +hZPE kJ mol^{-1}
39	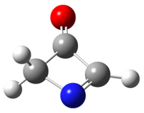	*3(2H)*-azetone	121.12	122.96
28	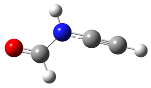	*t*-N-ethynyl-formamide	128.71	125.76
36	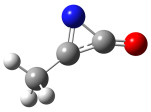	methyl-*2H*-azirin-2-one	127.93	126.03
10	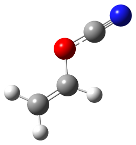	*t*-vinylcyanate	129.27	126.71
12	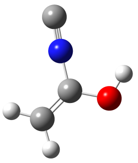	*g*-1-cyanoethenol	133.65	130.47
12b	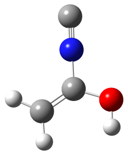	*t*-1-cyanoethenol	135.16	132.75
28b	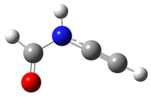	*g*-N-ethynyl-formamide	135.56	133.03
41	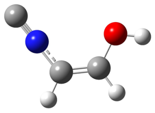	(*Z*)-*t*–isocyanovinylalcohol	137.17	136.70
37b	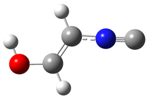	(*E*)-*g*-isocyanovinylalcohol	138.06	137.30

Continued on next page

TAG	Structure	Name	revDSD/junTZ kJ mol^{-1}	revDSD/junTZ +hZPE kJ mol^{-1}
37	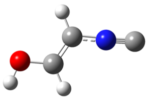	(*E*)-(*t*-isocyanovinylalcohol	139.63	138.09
15	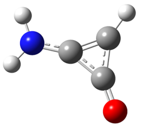	aminocyclopropenone	142.86	141.92
14	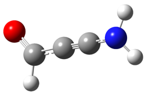	3-amino-2-propynal	153.98	151.84
17	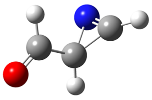	*2H*-azirine-2-carboxaldehyde	179.57	175.92
40	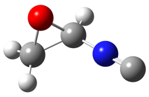	isocyanooxirane	200.48	201.19
30	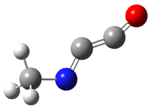	(methylimino)-ethenone	211.98	206.90
35b	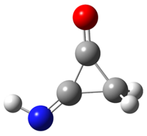	*g*-imino-cyclopropanone	224.85	219.81
35	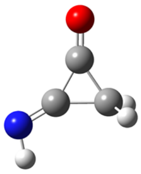	*t*-2-imino-cyclopropanone	228.16	222.94
34b	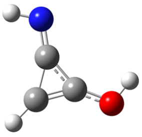	*tg*-3-imino-1-cyclopropen-1-ol	226.17	226.54

Continued on next page

TAG	Structure	Name	revDSD/junTZ kJ mol^{-1}	revDSD/junTZ +hZPE kJ mol^{-1}
34	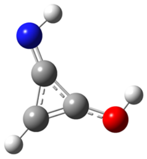	*tt*-3-imino-1-cyclopropen-1-ol	231.52	231.48
34d	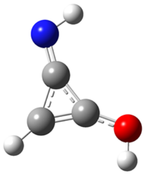	*gt*-3-imino-1-cyclopropen-1-ol	233.74	233.64
34c	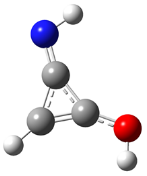	*gg*-3-imino-1-cyclopropen-1-ol	233.80	233.75
26	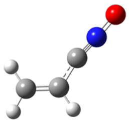	N-oxide-2-propenenitrile	245.57	242.76
21	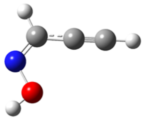	(*Z*)-*t*-oximepropynal	293.34	289.75
20	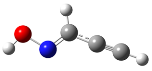	*E*)-*t*-oximepropynal	294.84	289.92
21b	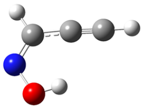	(*Z*)-*g*-oximepropynal	301.54	297.44
25b	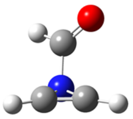	*g 1H*-azirine-1-carboxaldehyde	313.42	305.32
20b	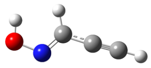	(*E*)-*g*-oximepropynal	317.53	311.42

Continued on next page

TAG	Structure	Name	revDSD/junTZ kJ mol^{-1}	revDSD/junTZ +hZPE kJ mol^{-1}
25	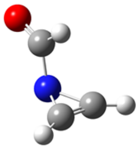	*t1H*-azirine-1-carboxaldehyde	322.28	312.64
42b	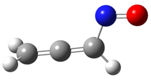	*t*-1-nitroso-1,2-propadiene	342.28	334.60
24b	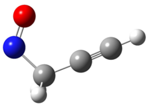	*g*-3-nitroso-1-propyne	362.86	354.94
24	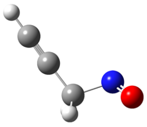	*t*-3-nitroso-1-propyne	365.25	357.62
38b	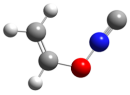	*g*-ethenylesterfulminicacid	365.97	361.24
38	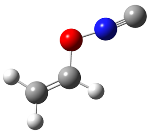	*t*-ethenylesterfulminicacid	374.69	368.20
31	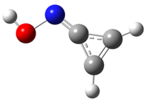	*t*-oxime-2-cyclopropen-1-one	384.37	379.56
31b	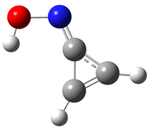	*g*-oxime-2-cyclopropen-1-one	406.00	399.79
19	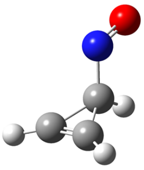	3-nitroso-cyclopropene	412.00	405.24

Continued on next page

TAG	Structure	Name	revDSD/junTZ kJ mol^{-1}	revDSD/junTZ +hZPE kJ mol^{-1}
42	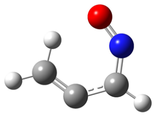	*g*-1-nitroso-1,2-propadiene	412.60	410.01
29	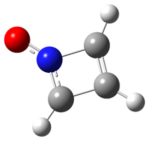	1-oxide-azete	435.06	431.13

2 CBS+CV energies

Table S2: CBS+CV and CBS+CV+aZPE relative energies (kJ mol^{-1}). For the sake of completeness, the harmonic (h) and anharmonic (a) ZPE corrections (Hartree, E_h / kJ mol^{-1}) at the revDSD/junTZ level are reported for the 10 species considered in the third step of the protocol.

TAG[a]	CBS+CV[b] kJ mol^{-1}	CBS+CV+aZPE[b] kJ mol^{-1}	hZPE E_h / kJ mol^{-1}	aZPE E_h / kJ mol^{-1}
5	0.00	0.00	0.05563 / 146.06	0.05505 / 144.53
	(-246.04806 E_h)	(-245.99301 E_h)		
1	-4.52	3.24	0.05867 / 154.04	0.05801 / 152.31
5b	3.04	3.31	0.05575 / 146.37	0.05515 / 144.80
3	8.14	4.50	0.05443 / 142.91	0.05366 / 140.88
6	11.97	9.42	0.05475 / 143.75	0.05407 / 141.96
6b	14.70	12.39	0.05478 / 143.82	0.05417 / 142.22
9b	12.41	14.05	0.05635 / 147.95	0.05567 / 146.16
8b	23.27	23.87	0.05597 / 146.95	0.05528 / 145.14
8	25.32	25.44	0.05573 / 146.32	0.05510 / 144.67
9	27.32	28.14	0.05595 / 146.90	0.05536 / 145.35

[a] For name and structure, see table S1; [b] Electronic energies obtained on top of the CBS+CV geometries.

3 CBS+CV optimised geometries

Table S3: CBS+CV optimised geometries for both forms of (E)-cyanovinylalcohol

(E)-t-cyanovinylalcohol			
Atom	x	y	z
N	0.0000000	0.0000000	0.0000000
C	0.0000000	0.0000000	1.1587452
C	-0.0145109	0.0000000	2.5819675
C	1.1352890	0.0000000	3.2636290
H	-0.9663516	0.0000000	3.0911541
H	2.0939068	0.0000000	2.7607069
O	1.1406919	0.0000000	4.6115518
H	2.0422119	0.0000000	4.9333403
(E)-g-cyanovinylalcohol			
Atom x	y	z	
N	0.0000000	0.0000000	0.0000000
C	0.0000000	0.0000000	1.1586396
X	2.5000000	0.0000000	1.1586396
X	2.5000000	0.0000000	-1.3413604
C	-0.0208713	0.0000000	2.5820404
C	1.1240751	0.0000000	3.2768878
H	-0.9858503	0.0000000	3.0701910
H	2.0894125	0.0000000	2.7925503
O	1.2302088	0.0000000	4.6151894
H	0.3564368	0.0000000	5.0150277

Table S4: CBS+CV optimised geometries for both forms of (Z)-cyanovinylalcohol

(Z)-t-cyanovinylalcohol			
Atom	x	y	z
N	0.0000000	0.0000000	0.0000000
C	0.0000000	0.0000000	1.1583546
C	-0.0314006	0.0000000	2.5824142
C	1.0814671	0.0000000	3.3240945
H	-0.9909728	0.0000000	3.0723610
H	1.0242367	0.0000000	4.4043414
O	2.3076281	0.0000000	2.7690079
H	2.9772163	0.0000000	3.4530907
Z)-g-cyanovinylalcohol			
Atom	x	y	z
N	0.0000000	0.0000000	0.0000000
C	0.0000000	0.0000000	1.1599289
C	0.1236256	0.0000000	2.5772942
C	1.3485413	0.0000000	3.1263356
H	-0.7582813	0.0000000	3.1943338
H	1.4916981	0.0000000	4.1960936
O	2.5068320	0.0000000	2.4571195
H	2.3366640	0.0000000	1.5069738

Table S5: CBS+CV optimised geometries for both isomers of cyanoacetaldehyde

t-cyanoacetaldehyde			
Atom	x	y	z
N	0.0000000	0.0000000	0.0000000
C	0.0000000	0.0000000	1.1554204
C	0.0559134	0.0000000	2.6136075
C	1.4896397	0.0000000	3.1231459
H	-0.4700219	0.8587054	3.0240633
H	-0.4294750	-0.9059958	2.9830425
H	2.2143767	-0.5737551	2.5237742
O	1.8083237	0.5704127	4.1304863
g-cyanoacetaldehyde			
Atom	x	y	z
N	0.0000000	0.0000000	0.0000000
C	0.0000000	0.0000000	1.1546641
C	-0.0236743	0.0000000	2.6118552
C	1.3572210	0.0000000	3.2387799
H	-0.5666894	0.8747792	2.9744405
H	-0.5666894	-0.8747792	2.9744405
H	1.3453827	0.0000000	4.3413898
O	2.3844737	0.0000000	2.6193834

Table S6: CBS+CV optimised geometries for both isomers of vinylisocyanate

t-vinylisocyanate			
Atom	x	y	z
O	0.0000000	0.0000000	0.0000000
C	0.0000000	0.0000000	1.1677025
N	0.1421103	0.0000000	2.3651561
C	-0.7104538	0.0000000	3.4709458
C	-0.2406495	0.0000000	4.7148482
H	-1.7707890	0.0000000	3.2579667
H	0.8204845	0.0000000	4.9120651
H	-0.9287009	0.0000000	5.5448429
g-vinylisocyanate			
Atom	x	y	z
O	0.0000000	0.0000000	0.0000000
C	0.0000000	0.0000000	1.1673203
N	-0.1412663	0.0000000	2.3646605
C	0.6961923	0.0000000	3.4825884
C	2.0269587	0.0000000	3.4409270
H	0.1551646	0.0000000	4.4159350
H	2.5691041	0.0000000	2.5059835
H	2.5869437	0.0000000	4.3625367

Table S7: CBS+CV optimised geometry of pyruvonitrile

pyruvonitrile			
Atom	x	y	z
N	0.0000000	0.0000000	0.0000000
C	0.0000000	0.0000000	1.1569168
C	-0.0436144	0.0000000	2.6391029
O	0.9840994	0.0000000	3.2649663
C	-1.4184994	0.0000000	3.2276144
H	-1.3487094	0.0000000	4.3102935
H	-1.9623411	-0.8778883	2.8793332
H	-1.9623411	0.8778883	2.8793332

4 Vibrational Spectroscopy: Vibrational Frequencies

Table S8: Harmonic and anharmonic vibrational frequencies (cm^{-1}) together with anharmonic intensities ($km{\cdot}mol^{-1}$) of pyruvonitrile and *g*-vinylisocyanate. Values computed at the revDSD/junTZ level.

	pyruvonitrile				*g*-vinylisocyanate			
	Harmonic	Anharmonic	I_{anh}	Exp.[a]	Harmonic	Anharmonic	I_{anh}	Lit.[b]
ν_1	3176.66	3035.85	3.1	3027	3270.44	3134.72	3.8	3140.2
ν_2	3053.65	2948.07	0.6	2924.5	3220.18	3108.91	3.1	3109.8
ν_3	2246.80	2210.91	37.0	2229	3170.06	3040.04	1.8	3045.1
ν_4	1770.45	1737.36	142.2	1740	2335.82	2282.90	594.8	2304.7
ν_5	1472.70	1434.78	14.7	1431.5	1683.05	1635.92	62.6	1663.6
ν_6	1405.34	1371.73	32.8	1368	1505.96	1482.88	17.0	1500.8
ν_7	1209.47	1179.06	105.5	1178	1437.91	1405.53	2.7	1419.2
ν_8	993.09	977.29	20.9	975.5	1337.85	1311.76	2.4	1324.9
ν_9	724.18	709.70	18.0	712	1090.64	1072.85	34.5	1085.1
ν_{10}	595.94	589.83	0.2	588.5	862.72	846.29	31.1	860.8
ν_{11}	433.1q	432.18	5.8	431	645.48	633.89	32.8	656.6
ν_{12}	176.99	177.09	8.8	176	494.13	492.46	16.6	494.3
ν_{13}	3124.06	2986.14	1.5	2975.5	117.85	113.11	4.2	110.1
ν_{14}	1480.68	1432.18	8.2	1428	1001.68	976.37	23.4	987.8
ν_{15}	1050.79	1028.80	4.3	1026	905.02	891.13	40.7	903.8
ν_{16}	589.62	584.86	0.7	535	697.94	687.01	4.6	690.9
ν_{17}	250.34	247.88	10.0	245	585.80	579.58	15.1	607.8
ν_{18}	136.34	131.25	0.1	-	110.07	99.52	2.2	90.0

[a] Low resolution experimental vibrational frequencies from ref. [1] [b] Theoretical CCSD/cc-pVTZ anharmonic frequencies from ref. [2]

Table S9: Harmonic and anharmonic vibrational frequencies (cm^{-1}) together with anharmonic intensities ($km \cdot mol^{-1}$) for both isomers cyanoacetaldehyde. Values computed at the revDSD/junTZ level.

	cyanoacetaldehyde							
	t				*g*			
	Harmonic	Anharmonic	I_{anh}	Lit.[a]	Harmonic	Anharmonic	I_{anh}	Lit.[a]
ν_1	3137.13	2996.23	0.1	3162.17	3058.56	2941.64	5.9	3128.09
ν_2	3061.77	2932.82	2.3	3083.76	2953.86	2839.85	65.2	2982.11
ν_3	2973.33	2854.65	38.8	2998.85	2293.56	2252.80	0.1	2204.54
ν_4	2284.93	2252.11	0.9	2197.36	1802.47	1769.94	100.9	1770.80
ν_5	1794.26	1765.29	147.7	1763.45	1449.64	1403.16	10.0	1451.03
ν_6	1451.80	1419.02	4.6	1455.85	1416.67	1384.64	8.2	1414.75
ν_7	1419.07	1385.48	6.4	1417.50	1339.98	1303.66	24.3	1333.07
ν_8	1297.34	1266.10	2.6	1291.59	1009.04	992.49	9.0	1012.43
ν_9	1227.92	1204.79	1.6	1229.95	849.70	822.29	10.9	855.64
ν_{10}	1050.03	1018.95	12.4	1055.48	730.08	718.08	20.1	728.69
ν_{11}	1046.07	1022.55	129.5	1043.33	392.62	391.10	5.6	384.76
ν_{12}	964.66	952.03	0.3	970.00	153.39	153.60	2.6	150.19
ν_{13}	737.87	708.58	2.2	739.87	3098.47	2957.61	0.0	3080.42
ν_{14}	521.08	516.01	0.5	516.58	1243.12	1215.16	0.3	1245.40
ν_{15}	469.15	463.96	13.4	466.13	1052.77	1031.92	0.0	1049.22
ν_{16}	351.33	345.66	0.2	343.08	722.02	709.56	1.2	718.72
ν_{17}	181.09	174.92	12.2	178.38	348.11	347.46	0.1	339.63
ν_{18}	61.23	46.81	21.5	63.14	130.12	131.82	0.6	133.97

[a] Harmonic frequencies at the MP2/aug-cc-pVTZ level from ref. [3]

Table S10: Harmonic and anharmonic vibrational frequencies (cm^{-1}) together with anharmonic intensities ($km \cdot mol^{-1}$) for both forms of (Z)-cyanovinylalcohol. Values computed at the revDSD/junTZ level.

	(Z)-cyanovinylalcohol							
	t				g			
	Harmonic	Anharmonic	I_{anh}	Lit.[a]	Harmonic	Anharmonic	I_{anh}	Lit.[a]
ν_1	3862.20	3678.44	124.9	3848.9	3740.55	3550.48	47.2	3715.8
ν_2	3239.61	3120.78	2.5	3261.2	3253.25	3118.84	3.5	3275.1
ν_3	3191.42	3017.27	4.0	3213.0	3228.53	3115.09	3.1	3249.6
ν_4	2260.37	2223.30	10.5	2179.3	2247.76	2210.54	16.2	2174.3
ν_5	1726.40	1682.95	83.4	1721.9	1682.68	1640.84	144.9	1683.2
ν_6	1437.49	1405.22	4.2	1431.7	1425.87	1393.73	5.2	1419.3
ν_7	1305.20	1286.17	192.6	1297.2	1381.36	1359.23	1.6	1374.1
ν_8	1256.99	1223.35	14.7	1248.7	1240.19	1214.06	71.3	1231.9
ν_9	1118.26	1093.95	28.3	1115.3	1108.89	1086.72	89.2	1103.7
ν_{10}	949.88	928.48	11.6	940.8	958.86	943.19	7.5	963.2
ν_{11}	728.59	727.68	15.3	724.6	741.56	732.39	4.1	739.7
ν_{12}	405.53	404.66	3.7	397.5	432.87	427.59	2.5	429.8
ν_{13}	149.37	156.02	1.0	145.2	134.83	133.12	8.3	129.42
ν_{14}	952.87	930.53	0.7	953.1	984.76	959.82	6.5	975.6
ν_{15}	744.54	739.00	43.6	742.5	734.24	725.87	33.5	733.9
ν_{16}	590.25	580.26	0.0	581.9	621.60	600.84	42.2	617.2
ν_{17}	371.86	364.33	72.0	368.1	521.02	503.31	52.2	517.9
ν_{18}	270.13	274.00	31.7	266.3	295.99	289.35	5.9	292.9

[a] Harmonic frequencies at MP2/aug-cc-pVTZ level from ref. [3]

Table S11: Harmonic and anharmonic vibrational frequencies (cm^{-1}) together with anharmonic intensities ($km \cdot mol^{-1}$) for both forms of (E)-cyanovinylalcohol. Values computed at the revDSD/junTZ level.

	(E)-cyanovinylalcohol						
	t			g			
	Harmonic	Anharmonic	I_{anh}	Harmonic	Anharmonic	I_{anh}	Lit.[a]
ν_1	3861.74	3677.68	136.4	3803.95	3621.64	66.3	3792.0
ν_2	3217.68	3094.67	4.0	3227.93	3097.21	4.3	3249.9
ν_3	3189.97	3072.17	3.8	3192.18	3062.02	5.5	3215.9
ν_4	2261.09	2226.68	19.2	2262.16	2224.52	21.2	2192.0
ν_5	1729.76	1683.26	110.4	1702.13	1658.52	192.2	1703.8
ν_6	1363.82	1349.24	31.0	1398.64	1373.36	8.4	1389.4
ν_7	1356.08	1312.13	19.6	1341.94	1318.33	16.7	1337.6
ν_8	1229.48	1199.77	220.4	1269.56	1240.07	42.0	1264.4
ν_9	1180.27	1152.32	14.0	1156.00	1133.30	204.0	1148.4
ν_{10}	1025.95	1006.99	3.4	1033.39	1034.84	15.5	1032.9
ν_{11}	559.12	552.91	5.8	554.57	548.48	1.1	549.9
ν_{12}	441.72	441.71	2.7	446.21	445.98	9.0	441.9
ν_{13}	177.49	177.18	7.0	177.94	178.78	7.9	174.1
ν_{14}	964.53	940.46	35.6	995.67	967.11	42.9	990.1
ν_{15}	828.99	817.11	15.1	807.53	793.24	30.3	803.8
ν_{16}	516.50	510.83	8.7	525.99	569.77	2.2	515.9
ν_{17}	363.54	358.00	95.7	481.26	383.90	72.1	472.5
ν_{18}	194.38	194.56	14.9	192.26	182.80	0.2	189.8

[a] Harmonic frequencies at the MP2/aug-cc-pVTZ level from ref. [3]

References

[1] H. Heise, F. Scappini, and H. Dreizler, "Acetyl cyanide III. vibrational spectrum and vibrational analysis," *Z. Naturforsch. A*, vol. 31, no. 11, pp. 1408–1412, 1976.

[2] K. Vávra, L. Kolesniková, A. Belloche, R. Garrod, J. Kouckỳ, T. Uhlíková, K. Luková, J.-C. Guillemin, P. Kania, H. Müller, *et al.*, "Millimeter wave spectrum and search for vinyl isocyanate toward sgr b2 (n) with alma," *Astron. Astrophys.*, vol. 666, p. A50, 2022.

[3] H. Møllendal, L. Margulés, R. A. Motiyenko, N. W. Larsen, and J.-C. Guillemin, "Rotational spectrum and conformational composition of cyanoacetaldehyde, a compound of potential prebiotic and astrochemical interest," *J. Phys. Chem. A*, vol. 116, no. 16, pp. 4047–4056, 2012.
